# Incidental discovery of invasive lobular carcinoma and anaplastic large cell lymphoma during sentinel lymph node biopsy: a case report

**DOI:** 10.3389/fonc.2024.1440126

**Published:** 2024-11-14

**Authors:** Euitae Kim

**Affiliations:** Department of Surgery, Dankook University College of Medicine, Cheonan, Republic of Korea

**Keywords:** lymphoma, large-cell, anaplastic, sentinel lymph node biopsy, case report, invasive lobular carcinoma

## Abstract

This study reports a rare case of concurrent invasive lobular carcinoma (ILC) of the breast and ALK-negative anaplastic large cell lymphoma (ALCL) detected during sentinel lymph node biopsy (SLNB). A 55-year-old female underwent breast cancer surgery following abnormal breast screening results, revealing ILC histologically. Unexpectedly, SLNB identified ALCL, later confirmed on pathology. The patient received no ALCL-specific treatment due to lack of additional lesions and remained recurrence-free post-surgery. The paper discusses diagnostic challenges, emphasizing comprehensive evaluation and multidisciplinary collaboration. It highlights the rarity of simultaneous ALCL and breast cancer without prior radiation, stressing the importance of clinical vigilance. Despite challenges in differentiation and treatment optimization, individualized patient care is crucial. Further research into concurrent ALCL and breast cancer is essential for improved management strategies. This case underscores complexities in managing rare malignancies and emphasizes the need for tailored approaches for optimal patient outcomes.

## Introduction

Breast cancer is the most common malignant condition in women globally as of 2020, with almost 2.26 million women newly diagnosed in that year. It ranks fifth among the most common causes of cancer-related death worldwide, yet it remains the leading cause of cancer death in less developed countries (1). Studies conducted by Gilchrist (2) and Zeidman and Buss (3) in the 1940s demonstrated that metastatic cells spread through regional lymphatics in an organized and reproducible manner, laying the groundwork for the evolution of Sentinel lymph node biopsy (SLNB). As a result, SLNB has become an integral part of contemporary breast cancer treatment, serving both as a staging and therapeutic tool, with axillary surgery traditionally considered essential (4). Anaplastic large cell lymphoma (ALCL) is a subtype of T-cell lymphoma (TCL) characterized by the presence of large cells and a strong, diffuse expression of the activation marker CD30. It is further classified into ALK-positive (ALK+) and ALK-negative (ALK-) subtypes based on the expression or absence of anaplastic lymphoma kinase (ALK) (5). Additionally, ALK-negative ALCL is currently categorized into systemic, primary cutaneous, and breast implant-associated ALCL (6). However, the simultaneous occurrence of ILC and ALK-negative ALCL is exceedingly rare and, to our knowledge, has not been previously reported. We aim to report on a case of ALCL incidentally detected during SLNB performed during breast cancer surgery in a patient who did not undergo breast augmentation with implants.

## Case description

**Figure 1:**
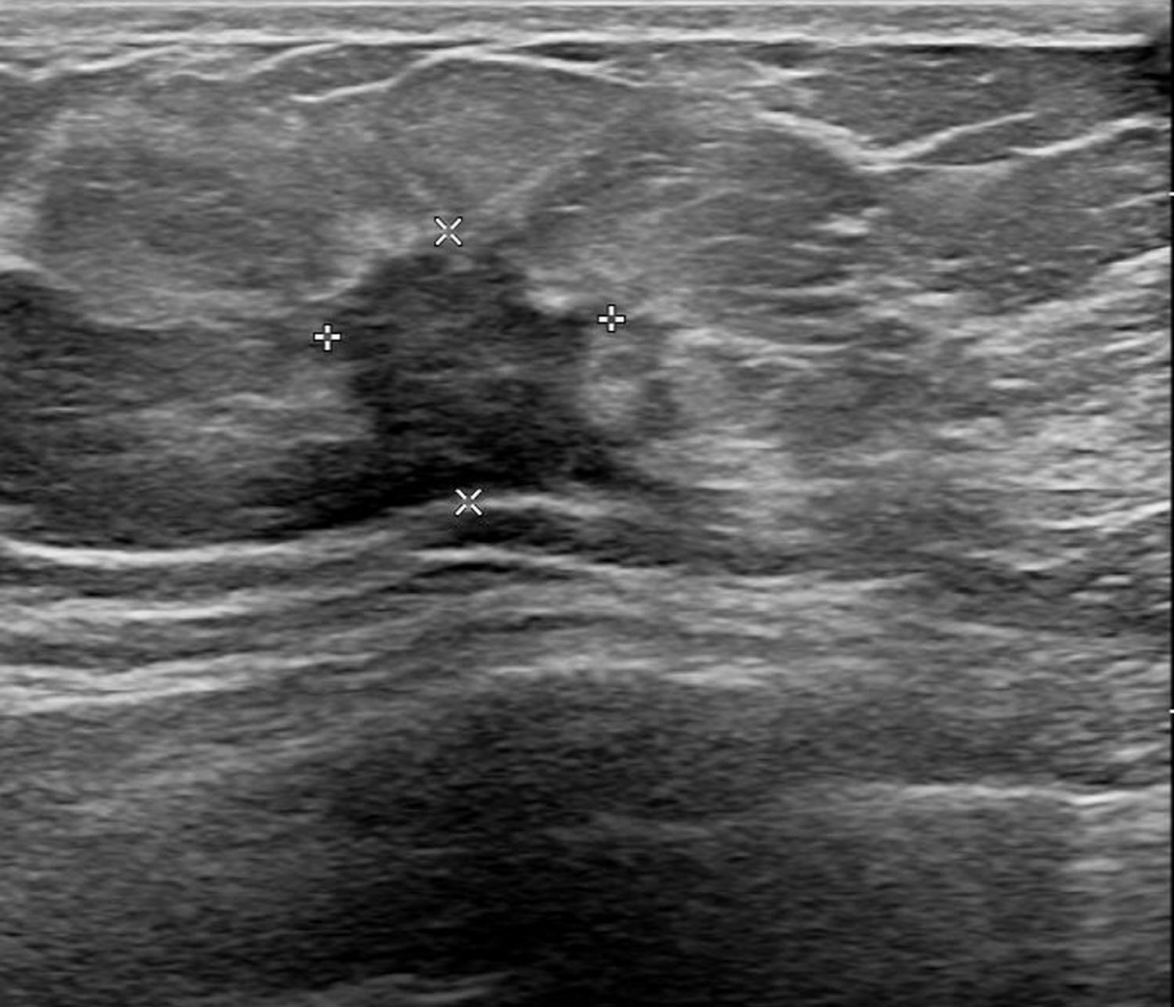
Ultrasound image of a breast lesion.

**Figure 2:**
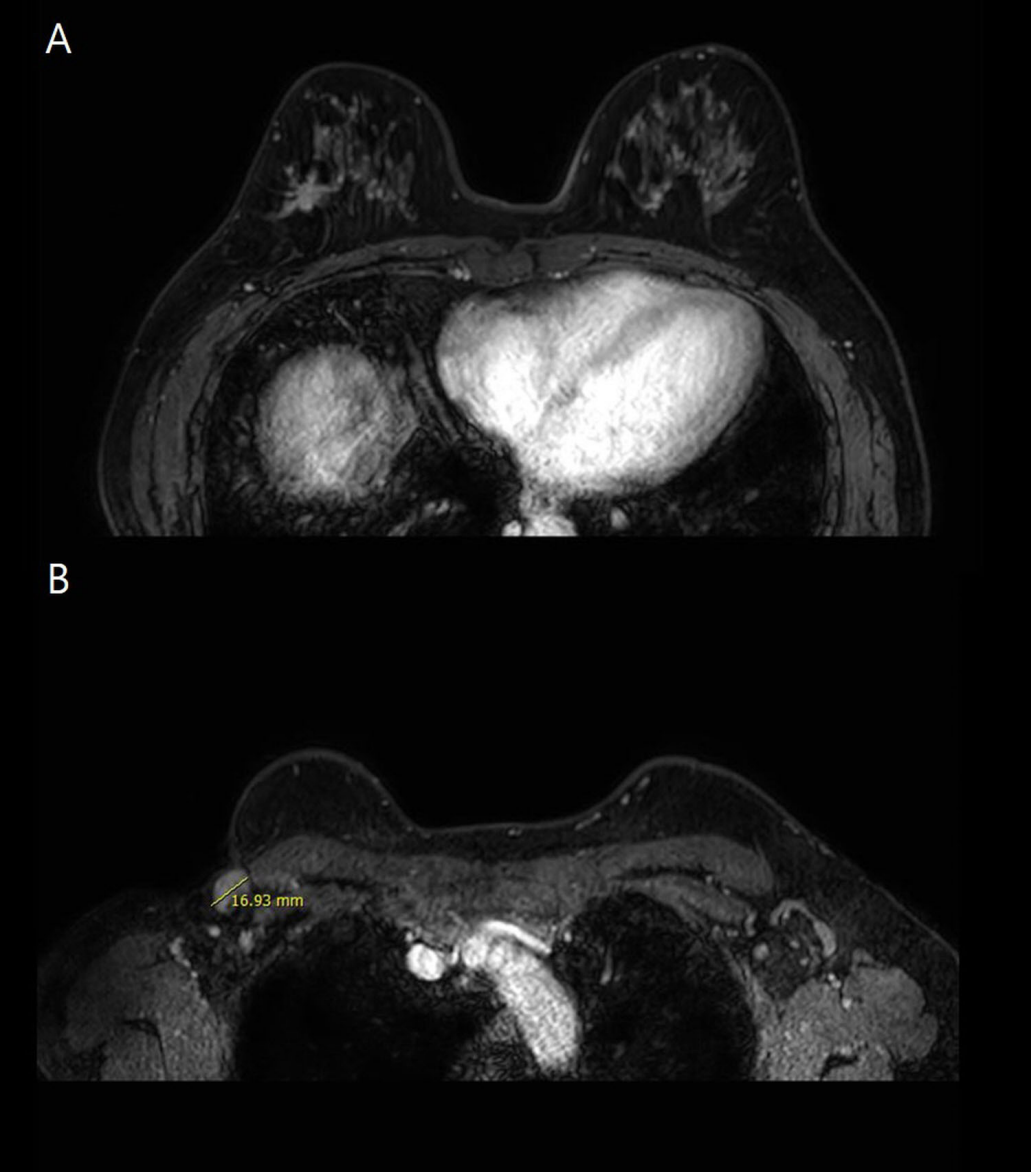
Breast MRI image of **(A)** breast and **(B)** axillary area.

**Figure 3:**
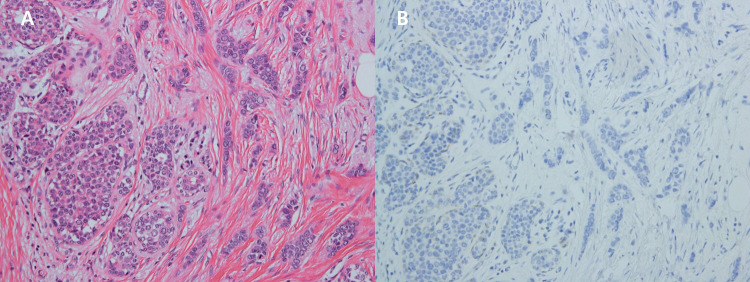
**(A)** x200 HE The breast tumor shows characteristic morphologies of invasive pleomorphic lobular carcinoma (right) and lobular carcinoma *in situ* (left). **(B)** x200 Immunohistochemistry Breast tumor cells are negative for E-cadherin.

**Figure 4:**
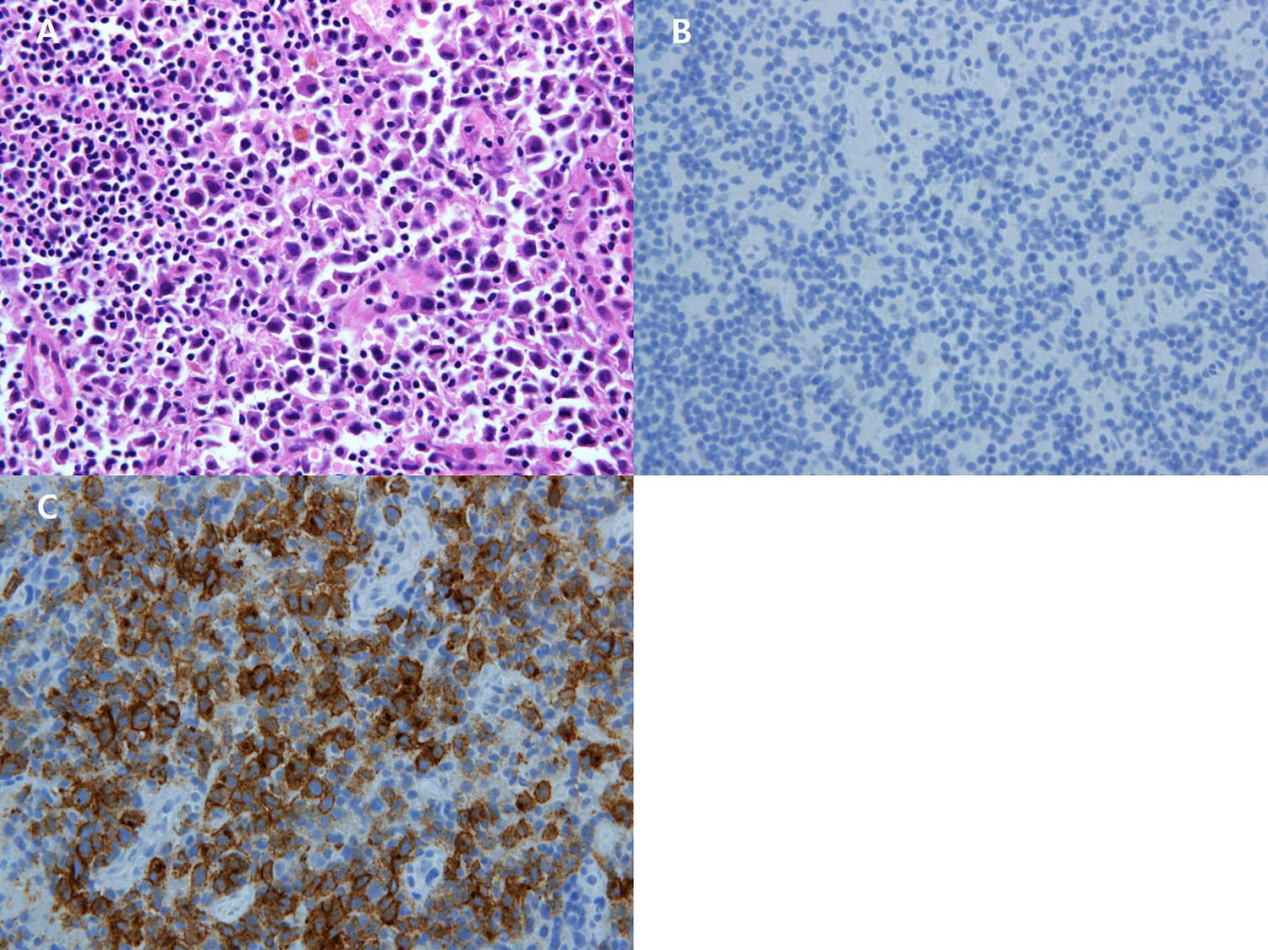
**(A)** x200 Immunohistochemistry Atypical large cells are positive for CD30. **(B)** x200HE The sentinel lymph node shows proliferation of large atypical lymphocytes within sinuses. **(C)** x200 Immunohistochemistry Large atypical cells are negative for cytokeratin.

A 55-year-old female patient visited the outpatient clinic after being recommended to undergo a tissue biopsy due to abnormal findings identified during regular breast screening mammograph (MMG) and breast ultrasound (US). The patient had previously undergone regular breast screening with MMG every two years, and no significant findings were observed during that period. She had no previous diagnosed conditions and was not taking any medications. The patient mentioned a family history of her father passing away from pancreatic cancer and her sister undergoing treatment for breast cancer. She started menarche at the age of 15 and is currently not in menopause. After the outpatient visit, an US was performed, revealing an indistinct margin hypoechoic mass measuring 1.5 cm, located 4 cm from the nipple at the 9 o’clock direction of the patient’s right breast (**Figure 1**). A core needle biopsy was performed on the detected mass, and the histological examination confirmed invasive lobular carcinoma (ILC). The immunohistochemistry staining results were estrogen receptor (ER) positive with an Allred score of 8, progesterone receptor (PR) positive with an Allred score of 8, human epidermal growth factor receptor-2 (HER2) score of 2, indicating equivocal result, and no amplification of HER2 was detected according to fluorescent *in situ* hybridization (FISH). To assess the staging of the patient before surgery, a breast magnetic resonance imaging (MRI), chest computed tomography (CT), abdominopelvic CT (A-P CT), and a bone scan were performed. The results of the examinations did not reveal any distant metastasis. The breast MRI showed a 2.1 cm x 1.1 cm x 2.2 cm lesion that was confirmed ILC (**Figure 2A**), and a suspicious lymph node measuring 1.7 cm was identified at axillary level I (**Figure 2B**). Additionally, a 0.5 cm low echoic lesion was observed in the 1 o’clock position of the right breast on breast US, and it was decided to remove it concurrently during the breast cancer surgery. Three weeks after the outpatient visit, the patient underwent a right breast lumpectomy and a SLNB. During the surgery, two lymph nodes were harvested for SLNB and they were examined using frozen section biopsy, which confirmed metastasis. As a result, an axillary lymph node dissection was performed for levels I and II of the axillary lymph nodes. The final pathologic report revealed that the breast cancer measured 3cm x 3cm in size ILC. The nuclear grade and histologic grade were both grade 1, indicating a well-differentiated tumor. Lobular carcinoma *in situ* was extensively present. Lympho-vascular invasion was not identified, and there was no lymph node metastasis among the 15 axillary lymph nodes examined, including the 2 sentinel lymph nodes. The hormone receptor status showed that the ER and PR were positive with an Allred score of 8. However, the HER-2 score was 1, indicating a negative result. The p53 protein was negative, suggesting no abnormalities, and the Ki-67 proliferation index was 10%, indicating a low level of cell proliferation. Interestingly, the final histological examination results differed from the intraoperative frozen section analysis as no evidence of breast cancer metastasis was found in the axillary lymph nodes. Enlarged sentinel lymph nodes were examined for pathologist’s intraoperative consultation. At that time large atypical cells were noted in the sinuses of the sentinel lymph nodes and it was difficult to distinguish metastatic lobular carcinoma from malignant lymphoma. Thus, the final decision for the sentinel lymph nodes was deferred to permanent sections. The lumpectomy specimen of the breast revealed typical microscopic features of invasive lobular carcinoma with associated extensive lobular carcinoma *in situ* (**Figure 3A**). Immunohistochemically the tumor cells revealed lack of E-cadherin expression (**Figure 3B**) and diffuse strong positivity for ER and PR. Therefore, the breast lesion was diagnosed as invasive lobular carcinoma. But the microscopic features of sentinel lymph nodes revealed proliferation of atypical large cells within sinuses (**Figure 4A**). Immunohistochemically the atypical cells were negative for cytokeratin (**Figure 4B**) and ER. Thus, the possibility of metastatic carcinoma was excluded. The atypical large cells in the sentinel lymph nodes were positive for CD45, CD30(**Figure 4C**), and MUM1, and negative for ALK, CK-multi (AE1/AE3), ER, CD10, EBV (*in situ* hybridization), CD15, EMA, granzyme B, TIA-1, p53, PAX-5, and CD20 (L26). The Ki-67 labeling index was high, and focal positivity was noted for CD3, CD2, CD4, CD5, CD8, CD45RO (UCHL1), and CD43. CD68 positivity was observed in sinusoidal histiocytes, while PR, CK5/6, E-Cadherin, p63, and bcl-2 were non-contributory. These findings led to the final diagnosis of ALK-negative ALCL in the sentinel lymph nodes.

Based on these final pathological results, the patient was diagnosed with both breast cancer and ALCL. The patient was referred to the hemato-oncology department for further testing and confirmation of ALCL. No additional lesions of ALCL were found in the chest CT and A-P CT performed as part of the preoperative evaluation. As adjuvant radiation therapy was planned to include the surgical site in the right axillary area, it was decided to observe the patient’s progress without additional treatment for ALCL. Six months after completing radiation therapy, follow-up examinations—including breast MRI, chest CT, and A-P CT—did not reveal any evidence of breast cancer or ALCL recurrence. Consequently, the decision was made to continue observation at six-month intervals while the patient received ovarian function suppression injections and tamoxifen as adjuvant treatment for breast cancer. Over the subsequent four years, no specific findings of recurrence or progression were observed, and the patient remains under ongoing surveillance.

## Discussion

Axillary nodal involvement has been firmly established as a prognostic indicator, with a decrease in 5-year survival ranging from approximately 28% to 40% in affected patients (7) Therefore, axillary surgery serves not only as a staging tool but also contributes to locoregional control, potentially leading to improved survival outcomes. The role of axillary surgery in clinically node-negative (cN0) axilla was initially investigated in trials conducted by the National Surgical Adjuvant Breast and Bowel Project (NSABP B-04) and the Cancer Research Campaign Working Party (King’s/Cambridge) (8, 9). These trials demonstrated that treatment of cN0 axilla with either surgery or radiotherapy did not confer a survival benefit compared to observation and treatment at the time of recurrence. As a result, SLNB has emerged as a guiding tool for determining disease staging and the need for axillary lymph node dissection.

It is already known that the incidence of breast cancer in young female patients increases after treatment of lymphoma due to chest radiotherapy performed for the treatment of Hodgkin lymphoma (HL) (10, 11). However, it is very rare that invasive breast cancer and axillary lymphoma are simultaneously identified in patients with no previous history of radiation therapy. In a previous literature report, there have been several reports of focal lymphoma in sentinel lymph node biopsy or axillary lymphoma *in situ* performed during surgery in patients with invasive breast cancer or ductal carcinoma *in situ*, but no reports of ALCL been identified as in this case (12–14).

The co-occurrence of breast cancer and lymphoma is a rare clinical scenario, one that remains poorly understood by oncologists, hematologists, and pathologists alike. In this case, the patient presented with ILC of the breast and ALK− ALCL, an uncommon combination. ILC itself is a less frequent subtype of breast cancer, often associated with a diffuse, infiltrative growth pattern and later-stage diagnosis (1, 2). ALK− ALCL, on the other hand, is a rare and aggressive form of lymphoma that typically affects older adults, with a median age of diagnosis in the sixth decade of life (5, 16). The patient in this case fits within the expected age range for both diseases, which may point to an age-related vulnerability or a potential shared mechanism, such as immune dysregulation, leading to the development of both malignancies.

ALCL is a type of TCL characterized by large cells and strong CD30 expression. It is divided into ALK+ and ALK- groups, with each comprising about 50% of cases. ALK+ ALCL is more common in children and has a better prognosis, while ALK- ALCL is more common in adults and has a more aggressive behavior (15). Diagnosis of ALK- ALCL can be challenging due to its morphological similarity to other large TCL with CD30 expression. Recent molecular studies have identified diverse genetic alterations in ALK- ALCL, leading to potential further subtyping based on genetic abnormalities (1, 15).

Differentiating between ALK- ALCL and carcinomas can be challenging due to potential morphological overlap, particularly in poorly differentiated cases. Carcinomas lack “hallmark” cells and exhibit glandular or squamous differentiation, distinguishing them from ALCL. ALK- ALCL is negative for cytokeratins and germ cell markers OCT3/4 and SALL4. Poorly differentiated carcinomas are cytokeratin-positive and lack CD30 and T-cell markers, with variable expression of organ-specific transcription factors. Careful interpretation of epithelial membrane antigen (EMA) is warranted, as it may be positive in ALK- ALCL (5). As a result, it is deemed that reaching a definitive conclusion from intraoperative frozen section pathology results would have been difficult.

Differentiating ALCL from other CD30+ lymphomas, such as HL, PTCL, and Mycosis Fungoides (MF), is critical due to differences in treatment approaches and prognosis. ALCL, characterized by large anaplastic cells expressing CD30, can be divided into ALK+ and ALK- subtypes, with distinct clinical and pathological features (5). HL, another CD30+ lymphoma, typically presents with Reed-Sternberg cells in a mixed inflammatory background. While CD30 expression is a common feature, ALCL differs by the presence of hallmark cells and the potential for ALK expression, which is absent in HL. Additionally, ALCL usually exhibits a more aggressive clinical course than HL, necessitating a different therapeutic strategy (6). PTCL is a heterogeneous group of T-cell malignancies, some of which can express CD30. However, unlike ALCL, PTCL lacks the characteristic large anaplastic cells with horseshoe-shaped nuclei. Immunophenotypic differences, such as the lack of ALK expression and distinct genetic aberrations, further aid in differentiating PTCL from ALCL (15). MF, primarily a cutaneous T-cell lymphoma, can exhibit CD30 positivity, particularly in transformed cases or in Sézary syndrome. However, MF typically presents with skin lesions and follows an indolent course, contrasting with the often systemic and aggressive nature of ALCL. The histopathological examination in MF shows epidermotropism and smaller, cerebriform lymphocytes, which are distinct from the large anaplastic cells of ALCL (16). In summary, while CD30 expression is a shared feature among these lymphomas, the diagnosis of ALCL is supported by its unique cellular morphology, the potential presence of ALK expression, and its distinct clinical presentation. Accurate differential diagnosis is essential for guiding appropriate therapeutic decisions and improving patient outcomes.

The management of ALK- ALCL poses challenges similar to those encountered in ALK+ ALCL. Despite higher relapse rates, current frontline therapies, primarily CHOP regimen (cyclophosphamide, hydroxydaunorubicin, oncovin, prednisone), yield unsatisfactory results with 5-year progression-free survival rates ranging from 30% to 55% (16). In most cases of follicular lymphoma that was concurrently diagnosed with breast cancer, adjuvant chemo therapy was administered (12). However, in the patient reported by Cox et al., who had low-grade follicular lymphoma, observation without adjuvant chemo therapy was also pursued (17). In our case, apart from ALCL identified solely in the axillary lymph nodes, no other suspicious lesions were found, and the patient did not exhibit any specific symptoms, so additional treatment for ALCL was not administered.

Recent advancements in molecular diagnostics, such as testing for DUSP22 and TP63 rearrangements, could offer valuable insights into ALK-negative ALCL prognosis and aid in distinguishing it from peripheral T-cell lymphoma (PTCL) NOS (5, 15). Although these tests were not performed in this case due to the lack of systemic lymphadenopathy and the stable condition of the patient, they might be considered in future cases to refine diagnosis and treatment plans.

## Conclusion

In conclusion, our report highlights a rare case of concurrent ILC of the breast and ALK- ALCL detected incidentally during SLNB. This underscores the necessity of thorough diagnostic evaluation and multidisciplinary collaboration in managing complex malignancies. The concurrent presence of ALCL and breast cancer without prior radiation therapy underscores the need for heightened clinical suspicion and comprehensive pathological assessment to ensure accurate diagnosis and appropriate treatment planning. The rarity of simultaneous ALCL and breast cancer without prior radiation therapy underscores the importance of heightened clinical suspicion and comprehensive pathological assessment to ensure accurate diagnosis and appropriate treatment planning. Challenges persist in differentiating ALCL from other large TCL and optimizing treatment strategies for ALK- ALCL. While our case contributes to existing literature, further research into the molecular characteristics and prognostic implications of concurrent ALCL and breast cancer is warranted to guide future management decisions. This case exemplifies the complexities inherent in managing rare malignancies and emphasizes the importance of individualized patient care and a multidisciplinary approach to optimize outcomes. Further research into the molecular characteristics and prognostic implications of concurrent ALCL and breast cancer is warranted to guide future management decisions.

## Data Availability

The datasets presented in this article are not readily available because nothing to restrict. Requests to access the datasets should be directed to EK, ket799@naver.com.
